# A Letter to the Editor: The Peer Review Process: Past, Present, and Future

**DOI:** 10.3389/bjbs.2024.14125

**Published:** 2025-01-15

**Authors:** Ahmed A. Khalifa

**Affiliations:** Orthopaedic Department, Qena Faculty of Medicine and University Hospital, South Valley University, Qena, Egypt

**Keywords:** research integrity, peer review, double blind, preprints, anonymous peer review

## Introduction

Dear editor, I have read the interesting article “The Peer Review Process: Past, Present, and Future” by Drozdz and Ladomery [1]. The authors elegantly describe the various approaches to peer review during scientific article evaluation and how each approach has its advantages and disadvantages.

The authors alluded to “Double anonymous peer review,” which is characterized by “Neither party knows the identity of the other at any point during the process [2],” as they referred to in their detailed review. Furthermore, they mentioned that one of the claimed benefits of this approach for a peer-review process is the reduction of possible biases originating from revealing the author’s identity (gender, affiliation, and other possible identities that could induce unconscious bias) [2–5].

## What Is Missing in the “Problems Associated With the Peer Review Process” Section?

I was expecting the authors to discuss how “Double anonymous peer review” is breached by the authors (intentionally or unintentionally) under the section Problems Associated With the Peer Review Process, which might include one or more of the following:

1- Some journals oblige authors submitting randomized controlled trials (RCTs) to provide the identity of their trial registration in one of the online registration databases (such as https://clinicaltrials.gov/). If the registration ID was presented in the “anonymized” or “blinded” version of the manuscript, the reviewers could quickly identify the authors and their details by checking the registration details.
2- Another example of breaching Double anonymous peer review occurs when authors submit a systematic review article. Reviewers could easily identify the authors if they reported details of their systematic review protocol registration (in one of the databases such as PROSPERO, https://www.crd.york.ac.uk/prospero/).
3- Some journals offer the authors the option to post their submitted, pre-peer-review manuscript to one of the preprint online repositories (such as Research Square https://www.researchsquare.com/), where the details of the authors are revealed [6].
4- If the authors state the setting of their study or define the details of their ethical committee board, their nationality or affiliation (not necessarily the exact authors’ identity) could be exposed.
5- Lastly, if the authors alluded to some of their previous work, such as mentioning “in a previous study, we reported….” The reviewer could identify the authors or at least the study group by following their previously published article.

## Discussion

In a scientific community overwhelmed by fraud, falsification, and questioned integrity, all possible actions must be followed to eliminate or minimize these dangers; one way to approach a fair and intact scientific research community is a sound peer review process, as explained in detail by Drozdz and Ladomery [1].

Although many peer review practices were suggested, and each has pros and cons, many scientists believe that the double anonymous peer review approach offers less bias as the reviewers do not know the author’s identity and *vice versa*. However, if it is breached by one of the abovementioned possibilities, it loses its main advantage.

Cooperation between the authors, the editorial team, and the reviewers is needed to ensure the integrity of the double-anonymous peer review process as follows:

1- The authors must follow the journal instructions and remove any information that could lead to their identification. They must also report if their manuscript was deposited in one of the preprint servers.
2- The editorial team should review the manuscript thoroughly to ensure an anonymous presentation. Further, some journals stated they would not accept submissions in which the original manuscript was published as a preprint [7].
3- The reviewers should decline to review manuscripts if the authors’ identity could be revealed. However, if they decide to complete their review process, they need to clearly state that they detected a breach regarding the blinding of the manuscript they had reviewed. In this situation, it is up to the editor to include their report or not.

In conclusion, the double-anonymous peer review approach is vulnerable to breaches, which could be intentional or unintentional. To improve the efficiency of such a process, cooperation between the authors, editorial team members, and potential reviewers is needed to detect and inform about breaches of the manuscript’s anonymized presentation.

## Data Availability

The original contributions presented in the study are included in the article/supplementary material, further inquiries can be directed to the corresponding author.
